# Publisher Correction: Comparison of two different doses of bleomycin in electrochemotherapy protocols for feline cutaneous squamous cell carcinoma nonsegregated from ultraviolet light exposure

**DOI:** 10.1038/s41598-021-88998-8

**Published:** 2021-05-03

**Authors:** Denner S. Dos Anjos, Oscar R. Sierra, Enrico P. Spugnini, Andrigo B. De Nardi, Carlos E. Fonseca-Alves

**Affiliations:** 1 Department of Veterinary Clinic and Surgery, São Paulo State University (UNESP), Jaboticabal, Brazil; 2 Biopulse srl, Via Toledo 256, 80132 Naples, Italy; 3 Department of Veterinary Surgery and Animal Anesthesiology, São Paulo State University - UNESP, Botucatu, SP Brazil; 4 Institute of Health Sciences, Paulista University-UNIP, Bauru, SP Brazil

Correction to: *Scientific Reports* https://doi.org/10.1038/s41598-020-75472-0, published online 27 October 2020

Due to a technical error the Supplementary Information file accompanying the Article contains a duplication of the Article contents.

The correct Supplementary Information file is provided below.

**Additional information**

**Supplementary Information** The online version contains supplementary material available at 10.1038/s41598-021-88998-8.

## Supplementary Information

Supplementary Information.

