# Supplementary material for: Comparison of two different doses of bleomycin in electrochemotherapy protocols for feline cutaneous squamous cell carcinoma nonsegregated from ultraviolet light exposure
Source: Sci Rep. 2020 Oct 27;10:18362. doi: 10.1038/s41598-020-75472-0 (PMC7591921; doi:10.1038/s41598-020-75472-0)
Supplement: Supplementary file 1 — Supplementary Information [file 41598_2020_75472_MOESM1_ESM.docx]

Comparison of two different doses of bleomycin in electrochemotherapy protocols for feline cutaneous squamous cell carcinoma nonsegregated from ultraviolet light exposure

Denner S. Dos Anjos^1*^, Oscar R. Sierra^1^, Enrico P. Spugnini^2^, Andrigo B. De Nardi^†1^, Carlos E. Fonseca-Alves^†^*^3,4^

^1^ Department of Veterinary Clinic and Surgery, São Paulo State University (UNESP), Jaboticabal, Brazil

^2^ Biopulse srl, Via Toledo 256, Naples 80132, Italy

^3^ Department of Veterinary Surgery and Animal Anesthesiology, São Paulo State University - UNESP, Botucatu-SP, Brazil.

^4^Institute of Health Sciences, Paulista University-UNIP, Bauru-SP, Brazil.

*Correspondence: [denner.anjosoncology](mailto:denner.anjosoncology)@gmail.com; +556798144-8191 and [carlos.e.alves@unesp.br](mailto:carlos.e.alves@unesp.br); +551499195-3871 (CEFA)

^†^These authors contributed equally to this work.

**Abstract:** Cutaneous squamous cell carcinoma (cSCC) is one of the most common skin tumors in cats due to chronic exposure to ultraviolet light. Local treatments such as electrochemotherapy (ECT) promote disease control or even complete remission. We hypothesize that cats could benefit from treatments using bleomycin at reduced dosages. A prospective nonrandomized single-blind study evaluated the clinical parameters, site lesion, staging, disease-free interval (DFI) and survival time by comparing the standard dose of bleomycin (15,000 UI/m²) (n=22) with a reduced dose (10,000 UI/m²) (n=34) in cats with cSCC that underwent ECT as the sole treatment modality. No statistically significant difference in DFI or overall survival was observed between the 2 groups. A higher DFI was found in cats with a small tumor size (less than 0.33 cm³) compared with that for cats with a large tumor size (P = 0.045). Furthermore, a reduced overall survival time for cats with a higher stage in the standard group SG (T3 and T4) (P = 0.004) was observed when compared to that for cats with a lower stage (T1 and T2). In conclusion, ECT using both doses of bleomycin may achieve the same response rate in terms of the overall response, DFI, and overall survival.

**Keywords:** bleomycin, cats, cutaneous carcinoma, electroporation, reduced dose, tumor size

**Introduction**

Cutaneous squamous cell carcinoma (cSCC) accounts for 10% of skin tumors in cats due to chronic exposure to ultraviolet (UV) light [1,2]. The lesions usually develop in pale or unpigmented skin with white areas being at the greatest risk [3]. Since fur is a physical barrier to UV radiation, the most commonly areas affected by cSCC are the ears, eyelids, nasal planum, lips and temporal areas [4-6]. Most of these tumors have an insidious progression characterized by nonhealing scabbing lesions that tend to progress over time to ulcers. As a result, these cats are mostly diagnosed in late stages, showing rapid tumor growth with advanced disease that requires aggressive treatment such as surgery combined with radiation therapy [1,7].

Among treatments indicated for cSCC (surgery, cryosurgery, radiotherapy, photodynamic therapy or medical management), surgical excision is the most successful way of treating advanced stage lesions (T3 or T4) of the pinna, eyelids, and nasal planum. However, the major limitation of surgery is the cosmetic outcome being unacceptable for some owners [1].

Chemotherapy has been used for controlling local disease or downstaging gross disease, but its use has been limited in feline cSCC due to the poor response rate [1,8-10]. However, the use of lipophobic agents has been used combined with electroporation, promoting cell death after enhancing the uptake of these drugs within neoplastic cells [1,6]. This therapy, known as electrochemotherapy (ECT), has been investigated over the past 15 years as an additional treatment modality for local control of solid tumors [4-6,11]. In the veterinary oncology field, it can be used as palliative, adjuvant, or neoadjuvant treatment or administered at the time of surgery (intraoperative ECT) [11].

ECT uses either bleomycin or cisplatin (both lipophobic agents), and its efficacy has been shown in different types of tumors with different outcomes [12]. Bleomycin has been considered the cornerstone drug for ECT since its uptake is facilitated after permeabilization of the cell membrane by electric pulses, resulting in cytotoxicity improvement by 700-fold [11]. Bleomycin fragments the DNA, which results in G2-M phase arrest of the neoplastic cycle [11,13-16].

In addition, studies have used different doses of bleomycin ranging between 15 to 30 mg/m² in cats diagnosed with cSCC with varied results [5,6,11]. In humans, a reduced dose has been proposed (10,000 UI/m²) to show the same efficiency as the standard dose (15,000 UI/m²) [17] in elderly patients with nonmelanoma head and neck skin cancer. Furthermore, other authors have also used a reduced dose of 10 mg/m² and found good results in humans [18]. Likewise, some countries have experienced a serious shortage of this drug secondary to decreased production by manufacturers. Since the reduced dosage presented good results in humans, we hypothesize that cats could benefit from treatments using the reduced dosage. Thus, this study aimed to evaluate the clinical parameters, site lesion, staging, disease-free interval and survival time by comparing the standard dose of bleomycin (15,000 UI/m²) with a reduced dose (10,000 UI/m²) in cats with cSCC that underwent ECT as the sole treatment modality.

Results

**Toxicity**

**Local toxicity – Standard group (SG)**

After ECT treatment (n=22), local adverse effects such as the development of crusts, hyporexia, ulceration, nasal edema/obstruction, palpebral edema, and sneezing were noted. In the SG, 12 cats had cSCC located on the nasal planum, but only two received tube feeding for 7-14 days post-ECT for nutritional support due to nasal edema/obstruction generating anosmia. In the remaining 10 cats with cSCC located on the nose, tube feeding was not based on the owners’ decisions, and transitory hyporexia for up to 7 days was observed in all of them. Supporting care including gentle force (hand) feeding was done by the owners. In other locations (periocular, auricular, peripalpebral, and lips), hyporexia was not observed among the cats (data not shown).

**Local toxicity – Reduced group (RG)**

As observed in the SG, local adverse effects (n=34) such as development of crusts, hyporexia, ulceration, nasal edema/obstruction, palpebral edema, and sneezing were noticed. In the RG, 21 cats had cSCC located on the nasal planum, but only one received tube feeding for 14 days post-ECT for nutritional support. In other locations (peripalpebral, auricular, periocular, auricular, jaw, and lips), hyporexia was not observed among the cats (data not shown).

**Systemic toxicity – Standard and reduced groups**

No systemic toxicity was observed in either group treated with ECT. However, one cat in the RG experienced sudden death 40 days post-ECT from a suspicious thromboembolism; we cannot infer if it was due to tumor lysis syndrome.

**Response to treatment**

**Standard group**: In the ECT SG group, 10/24 lesions had complete remission (CR) (41.6 %), 11/24 had partial remission (PR) (45.8%%) and 3/24 had stable disease (SD) (12.5%) at four weeks post-ECT. No progressive disease (PD) was observed. At the end of the observation period (10 cats excluded due to death and the remaining 14 cats alive), 10/14 lesions had CR (71.43%), 1/14 had SD (7.15%), and 3/14 had PD (21.42%).

**Reduced group:** In the ECT RG group, 16/42 lesions had CR (38 %), and 26/42 had PR (61.90%) at four weeks post-ECT. Neither SD nor PD was observed at four weeks post-ECT. At the end of the observation period (10 cats excluded due to death and the remaining 29 cats alive), 21/29 lesions had CR (72.41%), 3/29 had SD (10.34%), and 5/29 had PD (17.24%).

Altogether, CR was achieved in 26/66 (39.4%) lesions, PR in 37/66 (56%) lesions, and SD in 3/66 (4.5%) lesions at four weeks post-ECT. At the end of the study (20 cats excluded due to death and the remaining 43 cats alive), 31/43 lesions had CR (72%), 8/43 had PD (18.6%), and 4/43 (9.3%) had SD (Supplementary Table 1). Supplementary Figures 1 and 2 show the response to treatment of some cats in both groups before and after ECT treatment.

Among the 20 cats that died during follow-up, death in 13 cats was attributed to PD of cSCC, whereas the remaining cats died from other comorbidities (hypertrophic cardiopathy, chronic kidney disease, secondary tumors, thromboembolism, and unrelated causes).

In the SG, the median disease-free interval (DFI) and overall survival time corresponded to 240 days (60 – 485 days) and 300 days (65 – 610 days), respectively. In the RG, the median DFI and overall survival time were 210 days (30 – 395 days) and 210 days (40 – 485 days,) respectively.

No statistically significant difference in the Kaplan-Meier curve for DFI was observed between the SG and RG groups (P = 0.346) (240 and 210 days, respectively), nor was there a difference in overall survival (P= 0.689) (300 and 210 days, respectively) (Figure 1).


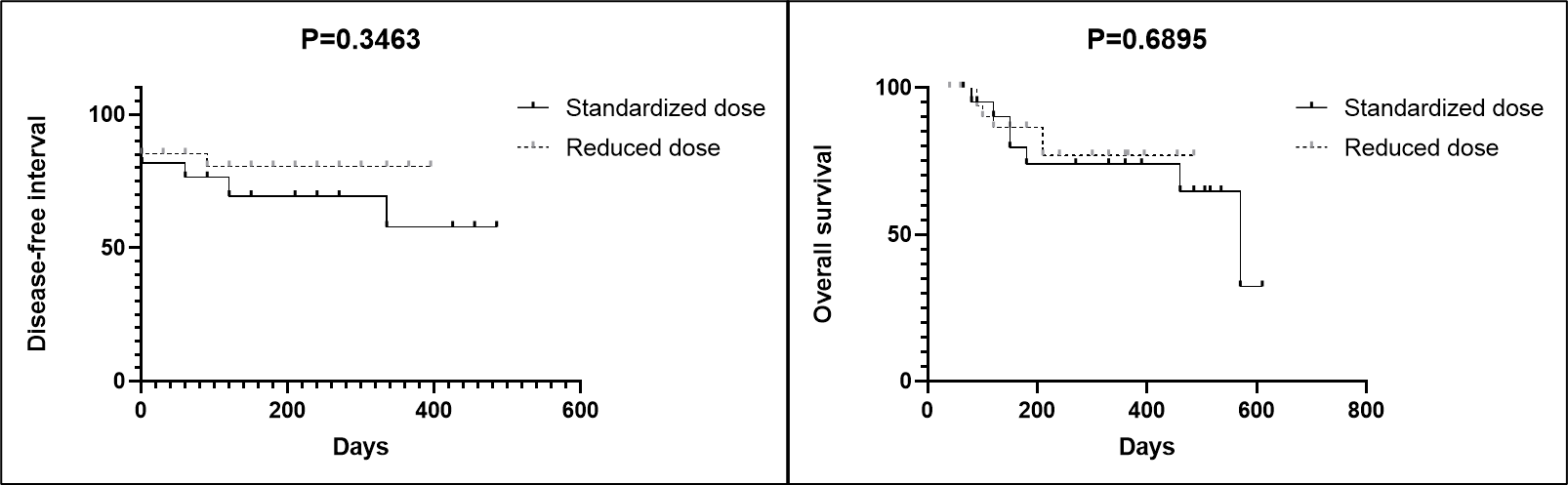


Figure 1. Kaplan-Meier curve for the disease-free interval and overall survival between the 2 bleomycin treatment groups of cats with cSCC that underwent ECT. No statistically significant difference in DFI (P = 0.346) (A) or overall survival (P= 0.689) (B) was observed between the 2 groups.

The cutoff for tumor size was based on the median of all lesions, which corresponded to 0.33 cm³. Then, tumors were classified as “low” when they were smaller than the median size, and “high” when they were larger than the median size.With regard to the impact of tumor size on DFI in the two cohorts in the RG and SG, we observed a significant difference in the former group (P = 0.045), with higher DFI in cat with a small tumor size (less than 0.33 cm³) compared with those with large tumor size (Fig. 2B).However, no statistically significant difference was observed between DFI and tumor size (P = 0.157) in the latter group (Fig. 2A).


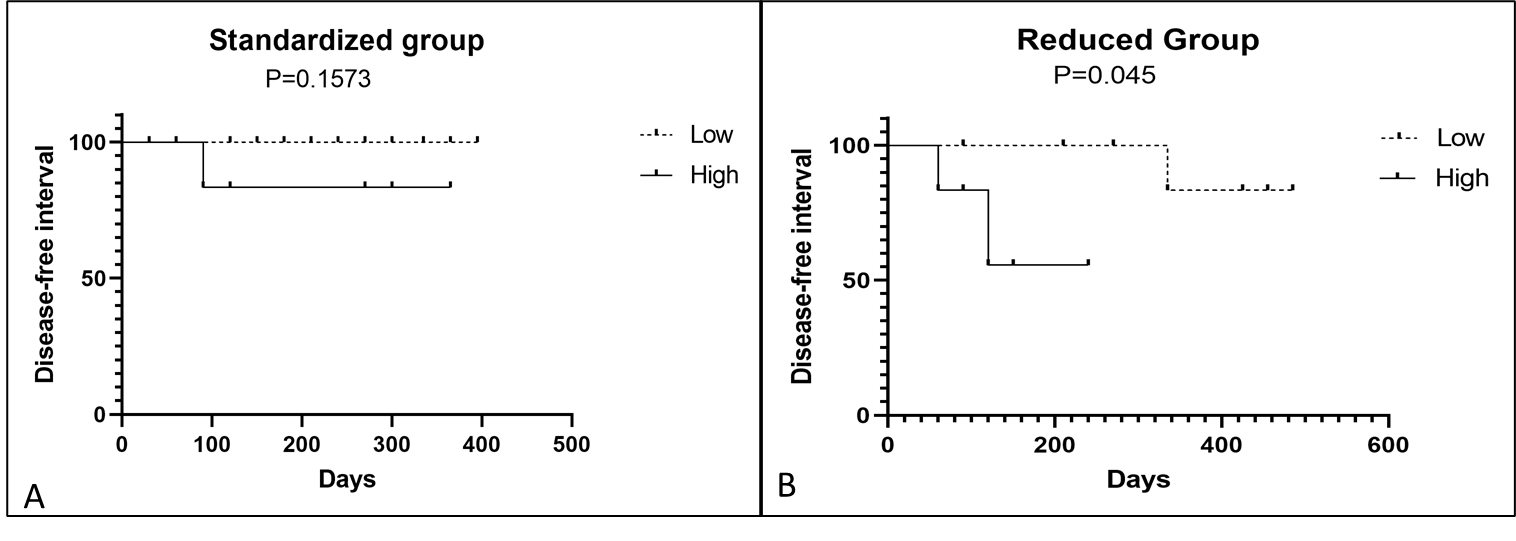
Figure 2. Kaplan-Meier curve for disease-free interval according to tumor size (cutoff of 0.33 cm³) between the 2 bleomycin-treated groups of cats with cSCC that underwent ECT. A statistically significance difference in the DFI according to tumor size was observed in the reduced group (P = 0.045) (B). In contrast, no statistically significant difference was observed in the DFI according to tumor size in the standardized group (P = 0.1573) (A).

The overall survival time was not statistically affected by tumor size in the SG or the RG (P = 0.661 (SG) and P = 0.265 (RG)) (Supplementary Figure 3 A, B). We observed a statistically significantly reduced overall survival time for cats with a higher stage in the SG (T3 and T4) (P = 0.004) when compared to cats with a lower stage (T1 and T2) (Fig. 3A). Nevertheless, in the RG, no statistically significant difference was observed in tumor stage (P = 0.495) (Fig. 3B).


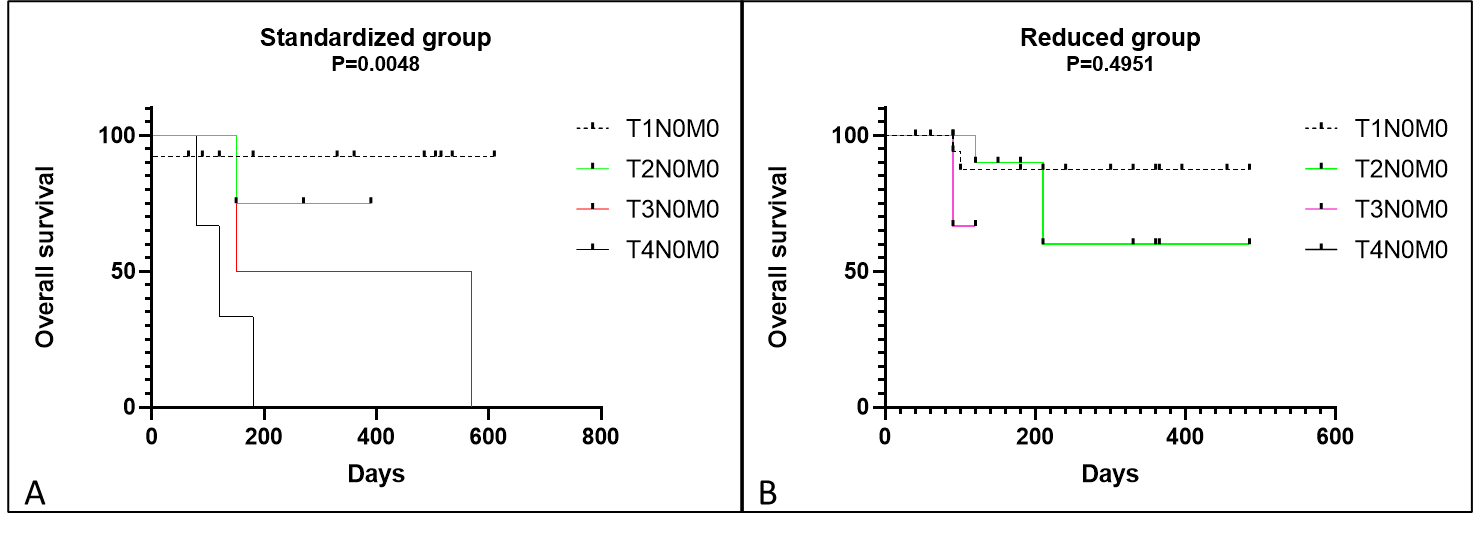


Figure 3. Kaplan-Meier curve for overall survival and tumor stage between the two groups of cats with cSCC that underwent ECT. A statistically significant difference in the standardized dose group was observed with a decrease in overall survival mostly in cats with more advanced stages of the disease (T3 and T4) (P = 0.004) (Fig. 4A). Nevertheless, in the reduced dose group, no statistically significant difference was observed between tumor stage and overall survival (P = 0.495) (Fig. 4B).

Based on the RECIST evaluation criteria, we evaluated the overall survival of patients that experienced CR, PR and SD at 4 weeks post-ECT. An expected statistically significant difference was observed in overall survival for cats that achieved a CR in the SG (P = 0.013). No difference was found in the RG (P = 0.191) (Figure 4A, B) (Supplementary Table. 1).


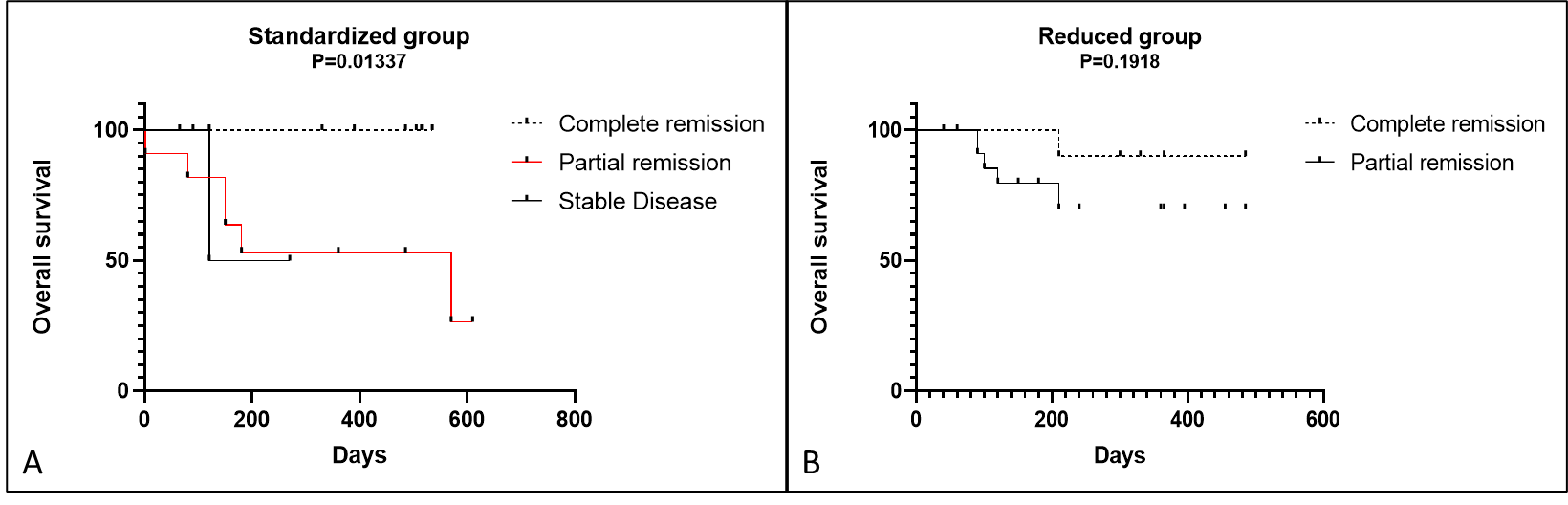


Figure 4. Kaplan-Meier curve for overall survival based on the RECIST criteria between the 2 groups of cats with cSCC that underwent ECT. A statistically significance difference was observed in the CR, PR and SD rates in overall survival (P = 0.013) (A). In contrast, no statistically significant difference was observed in the reduced dose group among CR and PR (P = 0.191) (B).

Considering the number of ECT sessions, we did not observe a statistically significant difference between the two groups (P > 0.05) (Supplementary Fig. 4). The median number of sessions in the SG and the RG was one (1-4) and one (1-2), respectively.

**Discussion**

This study demonstrated a similar efficacy between the SG and RG in terms of bleomycin treatment in cats with cSCC that underwent ECT without differences in DFI and overall survival. In addition, the ECT resulted in an overall response (CR + PR) of 95.4% at four weeks post-ECT. However, at the end of the observation period (1-20 months), the overall response rate was 72% (CR + PR), while the PD and SD rates were 18.6% and 9.3%, respectively. Notably, this clinical response may be due to possible residual neoplastic lesions in the site treated that progressed over time or due to persistent chronic solar exposure even after treatment (since all cats had outdoor access). In routine practice, most owners do not accept collection of a new biopsy after treatment, precluding the evaluation of residual lesions.

A previous report observed responses with ECT with CR in 81.8% (9/11) cats with cSCC and a DFI ranging from 2-42 months [5], similar to our results of a 72% CR rate (31/43) and a DFI ranging from 1-16 months. Another report also observed an overall response rate of 89% (CR + PR) among cats with acceptable adverse effects with a median time to progression of 30.5 months [6]. The treatment for cSCC is primarily surgical when feasible; however, to achieve clean surgical margins to gain good local control, an aggressive surgical excision is usually indicated [7, 19-21]. In a series report of 61 cats treated with surgery, radiotherapy, and cryosurgery, the surgery group had the longest median DFI of 19.8 months in cats with noninvasive disease [22]. However, a surgical approach is not always feasible due to the anatomical region involved, such as the nasal planum and eyelid. Previous reports observed a DFI of 12 months after reconstruction of the lower eyelid using a flap [7,21].

Furthermore, cats undergoing radiation therapy may suffer from adverse effects such as trichiasis and secondary corneal disease (ulcerative keratitis) [22,23]. In a study evaluating the efficacy of a hypofractionated radiation protocol for feline cSCC, 52% of cats responded to the treatment (40% CR and 12% PR) with a mean overall survival time and DFI of 224 and 271 days, respectively [24]. The efficacy rate of hypofractionated radiation in this previous study was much lower than that in cats in this study that underwent the ECT protocol with 72% responding to treatment (72% CR and 0% PR). However, the mean overall survival time (310 and 230 days in the SG and RG, respectively) and DFI (260 and 205 days in the SG and RG, respectively) were similar to those in our study.

In comparison with other nonsurgical approaches such as photodynamic therapy (PDT), a previous report observed a response rate of 85-90% in 55 cats with SCC, with a median time to recurrence of 157 days (109-205 days) [25]. This was a similar response rate to our response rate of 95.4% CR. However, PDT showed lower DFI results than those in our ECT study, which achieved a DFI of 210 days.

Based on this anatomical limitation and some secondary adverse effects of radiation therapy, one of the advantages of the ECT modality is its good antitumor efficacy, cosmetic effects and noninvasiveness, resulting in better outcomes in patients in whom radical surgery may not be an option [4-6, 11]. Previous studies have shown the efficacy of ECT treatment in incompletely resected soft tissue sarcomas in dogs and cats, showing a long DFI and improved local control when compared with surgery alone [26-30]. In this way, ECT coupled with bleomycin or using bleomycin and cisplatin simultaneously (injected intratumorally) may result in efficient tumor local control, and their use should be considered [29, 30].

After an extensive literature review of international databases, we found only one study with a considerable number of cats (47 cats) diagnosed with cSCC that were treated with ECT [6]; this study demonstrated a longer median time to progression of 30.5 months than that in our study (median time to progression of 7 months). In this previous study [6], the longer response could also be explained by the segregation of the cats according to the hours with the greatest ultraviolet (UV) exposure (11 AM until 4 PM). Unfortunately, in our study, cats were not segregated according to the timing of greatest UV exposure, which may have contributed to the lower DFI that that in the previous study. We suggest the possibility of using a UV screen in outdoor cats or retinoids or vitamin A supplementation as strategies to strengthen local control.

Other studies were identified; however, they presented few animals (9-11 cats,) [4,5]. In the preliminary study by Spugnini et al. [4], nine cats with SCC were enrolled and treated with ECT and showed a 77.7% CR rate. In the study by Tozon et al. [5], 11 cats were treated with ECT, showing an 81.8% CR rate (9/11) with a DFI of 2-42 months. In a nonrandomized prospective controlled study, Spugnini et al. [6] showed an overall response rate of 89% (23/26) in cats with advanced carcinoma of the head and periocular carcinoma in whom a surgical approach was not an option. In this study, patients who received a dose of 15,000 mg/m² intravenous bleomycin showed a long median time to progression of approximately 24.2 months (periocular cohort) and 20.6 months (advanced head SCC cohort). In our study, when we considered only patients with a tumor stage of T3 and T4 in both groups, we observed a better median overall survival in the SG of 150 days (80-540 days) and in the RG of 105 days (90-120 days) than in other research studies [4] that observed a DFI for cats with T3- and T4-stage disease (5/11) that ranged from 60-390 days. This reiterates the fact that ECT may be an alternative modality for the treatment of cats with advanced carcinoma.

Another point to highlight is the number of sessions cats underwent; most of them only underwent one session, and few cats received more than one session. In the ECT SG, 15/22 cats received one session, 5/22 received two sessions, 1/22 received three sessions, and 1/22 received four sessions, while in the ECT RG, 26/34 received one session, and 8/34 received two sessions.

The number of sessions could influence the response rates of the patients when considering the possibility of residual lesions. However, another study observed an 81.8% CR rate after a single ECT session [5], while Spugnini et al. [6] observed an 81% CR rate with the number of treatment sessionsranging from 2-9 (median, 4). When considering these findings, our CR rate was inferior to those from previous reports with a 50% CR rate with a single ECT session at the end of observation period.

Regarding the DFI according to tumor size, we observed a higher DFI in patients with a small tumor size in the RG (P = 0.045) but not in the SG (P =0.157). These results are expected since cats with a larger tumor size may have disease with a more infiltrative biological behavior, and they may have higher TNM staging [1]. The fact that the SG did not showed a significance difference may be due to the number of samples and heterogenous tumor sizes among cats, since the median tumor size when evaluate separatly in the SG was higher than that in the RG (0.815 versus 0.267 cm³). . The majority of publication on ECT used in humans and veterinary medicine oncology used different cutoff to evaluate effectiveness of ECT, i.e., tumors less than 3 cm size (cutaneous tumor) or less than 1cm³ (canine perianal tumors). Usually, in the different cutoff, it was observed a better tumor control and efficience of ECT in smaller tumors when compared with larger tumors [31-33]. However, since we prospectively included patients from routine practice, it was difficult to achieve a homogenous group in terms of tumor size. Thus, we opted to perfom a median value for all tumors (including animal from RG and SG) and used a median value as a cutoff value for bot tumors.

Another important aspect to consider is that, in our study, cats with large tumors (T3 and T4) showed decreased in overall survival when compared with those with small tumors (T1 and T2). This was also observed by Tozon et al. [5] and Spugnini et al. [6], who observed that cats with T3- and T4-stage disease showed poorer responses to ECT since these cats had highly invasive tumor growth. This poorer response may be due to bone microenvironment involvement as a cause of treatment failure since bone may act as a sanctuary for cancer cells and dictate tumor cell survival and recurrence [34,35].

Althought it was not a main goal of our research, clinical stage is an important prognostic factors for the treatment of feline cSCC, and this result was also found by other authors [4,6]. Furthermore, this difference was only noted in the SG compared with the RG; thus, this difference may be due to heterogenous stage numbers between the two groups. Further studies with a greater number of cats in each clinical stage could be important to affirm that clinical stage is strongly associated with patient prognosis. In the ECT SG, 13 cats had T1-stage disease (median DFI, 302 days), four had T2-stage disease (median DFI, 105 days), two had T3-stage disease (120 days), and three had T4-stage disease (no DFI observed). In contrast, in the ECT RG, 18 had T1-stage disease (median DFI, 225 days), 12 had T2-stage disease (median, DFI 90 days), three had T3-stage disease (no DFI observed), and one had T4-stage disease (no DFI observed).

One of the limitations of our study is that we did not have access to histological features or further biopsies after treatment to perform a comparison of histological changes. This approach could reveal some interesting information regarding what ECT with different dosages induces in tumor cells including the apoptosis rate, number of mitoses and tissue necrosis. However, due to complete remission in some cases and owner decisions in other cases, we were not able to perfom this evaluation. Previously, our research group observed that this therapy induced a decrease in the proliferation index and tumor volume 21 days after treatment in dogs with cSCC treated with ECT [36]; future studies evaluating tissue samples in feline cSCC could provide new valuable information.

We conclude that ECT with both doses of bleomycin may achieve the same overall response rate, DFI, and overall survival rate. In this way, ECT can be considered a potentially affordable alternative treatment for cats with cSCC even in advanced stages when surgery is not an option. Additional studies with a larger number of patients are warranted to elucidate an extended duration of local control, especially with biopsies taken after treatment to evaluate possible residual lesions.

Materials and Methods

Ethical approval. This study was performed in accordance with the National and International Recommendations for the Care and Use of Animals (National Research Council) [37]. This study was approval by the Ethics Committee on Animal Use (CEUA) of the Veterinary Teaching Hospital of University Estadual Paulista “Júlio de Mesquita Filho”, Jaboticabal, Brazil (CEUA/UNESP, protocol #016279/19).

Study design. To improve the precision of reported clinical studies on ECT, we strictly followed the design of Campana et al. [38], who developed 47 quality criteria clustered into four domains (trial design, description of the patient population, treatment delivery and outcome assessment and analysis of results and their interpretation. Furthermore, we also followed the checklist by Cemazar et al. [39] that outlines a proposal for proper nomenclature and guidance for reporting of materials and methods related to the use of electroporation of biological samples in in vivo applications.

This was a prospective nonrandomized single-blinded comparative clinical trial evaluating cats with cSCC that underwent ECT. Informed consent to perform the treatment was obtained from the cats’ owners. Follow-up was dated until the last examination of the patient with a minimum one-month follow-up. We defined the term “patients” as cats, and no human participants or human data were involved in the study.

Case selection criteria. All cats enrolled in this study had to fulfill the following criteria: cytologically (fine-needle aspiration) or histopathologically confirmed cSCC diagnosis (some cats lacked histopathological examinations); absence of distant metastases; compliance of the owner with follow-up after four weeks; normal physical exam; and clinical staging based on the tumor, nodes and metastasis (TNM) system from the World Health Organization [40].

Patients. A power analysis was performed to determine the minimum sample of patients to be enrolled for adequate study power. We used a type 1 error of 5% and a power of 80% and determined that at least 12 subjects would be needed in each group using a sample size calculator (<https://clincalc.com/stats/samplesize.aspx>). Then, between July 2018 and December 2019, 56 cats with cSCC were included. Six cats had more than one affected site for a total 66 lesions submitted to ECT. Twenty-two cats were included in the ECT standard group (SG) (15,000 UI/m²), and 34 cats were included in the ECT reduced group (RG) (10,000 UI/m²).

The allocation of subjects to treatment groups was accomplished by alternatively including the cats in each of the ECT groups (first in the SG, second in the RG, third in the SG, fourth in the RG, and so on). When we achieved a considerable number of cats in the SG (22 cats), we arbitrarily decided to include cats only in the RG afterwards until 34 cats in total were included (leading us to have a minimal follow-up of four weeks). We established an observational period of one moth (30 days) as the minimum observational period and 20 months the maximum observational period. All cats were mixed breed cats except for one Persian in the SG; their ages ranged from 4 to 17 years (median, 12 years) and from 5 to 20 years (median, 10 years) in the SG and RG, respectively. Feline immunodeficiency virus (FIV) and feline leukemia virus (FeLV) were not tested for in the included cats. There were 14 spayed females and 8 castrated males in the SG, whereas in the RG, there were 25 spayed females and 9 castrated males.

The most commonly affected sites observed in the SG were the nasal planum (n=12) followed by the palpebral region (n=5), labial region (n=3), periocular region (n=2), auricular region (n=1) and ocular region(n=1). In the RG, the most affected sites were the nasal planum (n=21) followed by the palpebral region (n=10), auricular region (n=5), periocular region (n=3), labial region (n=1), ocular medial canthus (n=1) and jaw region (n=1).

None of the patients had nodal metastasis at presentation. In the ECT SG, 13 cats had stage T1, four had stage T2, two had stage T3, and three had stage T4 disease. In contrast, in the ECT RG, 18 had stage T1, 12 had stage T2, three had stage T3, and one had stage T4 disease. The tumor size ranged from 0.02 cm³ to 28.8 cm³ in the SG (median, 0.815 cm³), whereas in the RG, the tumor size ranged from 0.0005 to 57.8 cm³ (median, 0.267 cm³).

In the SG, one cat had history of cryosurgery, and one had been treated with corticosteroids prior to referral, whereas the other cats had no prior treatments. In the RG, one cat had a history of two previous surgeries. Patients had received previous treatment approximately three to six months before ECT. Therefore, ECT was not considered adjuvant therapy. All neoplastic lesions had been present, accordingly to the cats’ owners, for at least 6 months before referral. No other concomitant oncological treatment was performed in cats from this study.

Electrochemotherapy protocol. We attempted to follow the updated standard operating procedure (SOP) for ECT of cutaneous tumors in humans and animals [41,42]. Bleomycin (Bleocris, Quality Pharma, LKM Laboratory, Argentina) was diluted in 5 mL of saline solution and administered intravenously (IV). The ECT protocol was divided into two groups: ECT SG (15,000 UI/m²) and ECT RG (10,000 UI/m²). In both groups, 5 minutes after the administration of bleomycin, one train of 8 biphasic pulses at a voltage per centimeter of 800-1,300 V/cm and 1-Hz frequency lasting 50 + 50 µs with a 300-µs interpulse (total treatment time per train, 3.2 ms) was administered by means of a certificated clinical electroporator for veterinary application (Onkodisruptor, Biopulse S.r.l., Naples, Italy) using a plate or a single pair of needle array electrodes until complete coverage of the lesion. The decision to use both a plate and needle was based on the tumor size; namely, larger volumes was treated with combination electrodes (> T2 stage). Good contact between the electrodes (plates) and the skin was assured by depilation and application of a conductive gel to the treatment area. The number of sessions ranged from 1-4 sessions (30-day interval between sessions). All ECT procedures were performed under general anesthesia; for induction, propofol was administered (Provive, União Química - Farmacêutica Nacional S/A. São Paulo, SP, Brazil) (5 mg/kg) followed by endotracheal intubation; anesthesia was maintained with isoflurane (Isoforine, Cristália Produtos Químicos Farmacêuticos Ltda. Itapira, SP, Brazil). All animals received postoperative analgesia including an IV injection of dexamethasone (Dexamethasone 0.2%, Hypopharma, Minas Gerais, Brazil) and tramadol (Tramal União Química - Farmacêutica Nacional S/A, São Paulo, SP, Brazil) (2 mg/kg).

Furthermore, all cats received a post-ECT oral antibiotic (amoxicillin trihydrate and clavulanate potassium at dosage of 20 mg/kg orally q12h), corticosteroids (prednisolone 1 mg/kg orally q24h) for one week due to ulceration and transitory edema and tramadol (3 mg/kg for three consecutive days q12h) for analgesia (Cronidor, Agener União, São Paulo, SP, Brazil).

Electrodes. These were either (1) Onkodisruptor M1 (M1 Clamp) adjustable clamp electrodes with a distance between 9 and 45 mm (we set them at 10 mm) between stainless-steel plates (dimensions: 10 mm width and 60 mm length) or Onkodisruptor spherical probes, which include a pair of two needle-shielded electrodes (80 mm length) with a special insulating resin shield that guarantees the application of voltage only in the last 5 mm of the electrode (https://www.onkodisruptor.com/electrode-holders/). Furthermore, two needle-shielded electrodes were inserted into the tumor while the clamp electrode was used superficially. A conductive gel was used for the M1 Clamp between electrodes to achieve good contact.

Tumor response. We estimated the tumor volume using the following formula: V=ab²π/6 (where “a” is the larger diameter of the tumor nodule, and “b” is the diameter of the tumor nodule perpendicular to “a”). Moreover, we used the Response Evaluation Criteria in Solid Tumors (RECIST) system to measure and assess the response of tumor lesions [43,44]. CR was defined as total reduction in the measured tumor volume, and PR was defined as a ≥ 30% reduction in tumor volume. PD was defined as a ≥ 20% increase in tumor volume or new lesions, and SD was defined as a ˂ 30% reduction in tumor volume or a ˂ 20% increase in tumor volume [39]. In cats that had more than one site lesion, the worst lesion (worst tumor stage) was considered for statistical evaluation.

Statistical analysis. For statistical purposes, we calculated the median value of each clinical parameter (tumor size and tumor stage) and classified the value as “low” when it was lower than the median and “high” when it was greater than the median. Then, we compared the survival rates of cats with low and high values of each parameter using the log-rank test and Kaplan-Meier curves. To evaluate the association of the tumor stage and DFI with survival time between the ECT standard and reduced groups, Fisher’s exact test was applied. For the survival curve, animals that died from other diseases or that were lost to follow-up were censored. When the subjects died from the disease, they were classified as uncensored. Commercial software (GraphPad Prism 8.1.0 – GraphPad Software) was used for the statistical analysis. P values <0.05 were considered significant.

References

1. Murphy, S. Cutaneous squamous cell carcinoma in the cat current understanding and treatment approaches. *J. Fel. Med. Surg*. 15, 401-407 (2013).

2. Hauck, M.L. & Oblak, M.L. Tumors of the skin and subcutaneous tissues. In Vail, D.M., Thamm, D.H. & Liptak, J.M. Small animal clinical oncology. 6th ed. St. Louis, Missouri, Elsevier, 2020; pp. 353.

3. Dorn, C.R., Taylor, D.O. & Schneider, R. Sunlight Exposure and Risk of Developing Cutaneous and Oral Squamous Cell Carcinomas in White Cats. J Natl Cancer Inst. 46, 1073-1078 (1971).

4. Spugnini, E.P. et al. Electrochemotherapy for the treatment of squamous cell carcinoma in cats: A preliminary report. *Vet. J.* 179, 117-120 (2009).

5. Tozon, N., Pavlin, D., Sersa, G., Dolinsek, T. & Cemazar, M. Electrochemotherapy with

intravenous bleomycin injection: an observational study in superficial squamous cell carcinoma in cats. *J. Fel. Med. Surg*. 16, 291-299 (2014).

6. Spugnini, E.P. *et al*. Electroporation Enhances Bleomycin Efficacy in Cats with Periocular Carcinoma and Advanced Squamous Cell Carcinoma of the Head. *J. Vet. Intern. Med*. 29, 1368-1375 (2015).

7. Schmidt, K., Bertani, C., Martano, M., Morello, E. & Buracco, P. Reconstruction of the Lower Eyelid by Third Eyelid Lateral Advancement and Local Transposition Cutaneous Flap After ‘‘En Bloc’’ Resection of Squamous Cell Carcinoma in 5 Cats. *Vet. Surg*. 34, 78-82 (2005).

8. Theon, A.P., VanVechten, M.K. & Madewell, B.R. Intratumoral Administration of Carboplatin for Treatment of Squamous Cell Carcinomas of the Nasal Plane in Cats. *Am. J. Vet. Res.* 57, 205-210 (1996).

9. Kisseberth, W.C. et al. Phase I Clinical Evaluation of Carboplatin in Tumor-Bearing Cats: A Veterinary Cooperative Oncology Group Study. J. Vet. Intern. Med. 22, 83-88 (2008).

10. Martinez-Ruzafa, I. et al. Tolerability of Gemcitabine and Carboplatin Doublet Therapy in Cats with Carcinomas. *J. Vet. Intern. Med*. 23, 570-577 (2009).

11. Spugnini, E.P. & Baldi, A. Electrochemotherapy in Veterinary Oncology State-of-the-Art and Perspectives. *Vet. Clin. Small Anim*. 49, 967-979 (2019).

12. Cemazar, M. & Sersa, G. Recent Advances in Electrochemotherapy. *Bioelechem*. 4, 204-213 (2019).

13. Spugnini, E.P., Azzarito, T., Fais, S., Fanciulli, M. & Baldi, A. Electrochemotherapy as first line cancer treatment: experiences from veterinary medicine in developing novel protocols. *Curr. Cancer Drug Targets*. 16, 16:43–52 (2016).

14. Spugnini, E.P., Fanciulli, M., Citro, G. & Baldi, A. Preclinical models in electrochemotherapy: the role of veterinary patients. *Future Oncol.* 8, 829–37 (2012).

15. Spugnini, E.P., Fais, S., Azzarito, T. & Baldi, A. Novel instruments for the implementation of electrochemotherapy protocols: from bench side to veterinary clinic. *J. Cell Physiol.* 232, 490–495 (2017).

16. Spugnini, E.P. *et al.* Definition of novel electrochemotherapy parameters and validation of their in vitro and in vivo effectiveness. *J. Cell Physiol*. 229, 1177–81 (2014).

17. Groselj, A. *et al*. Efficiency of electrochemotherapy with reduced bleomycin dose in the treatment of nonmelanoma head and neck skin cancer: Preliminary results. *Head and Neck*. 40, 120-125 (2017).

18. Mir, L.M. *et al*. Effective treatment of cutaneous and subcutaneous malignant tumours by electrochemotherapy. *Brit. J. Cancer*. 77, 2336-2342 (1998).

19. Hauck, M. L. & Oblak, M.L. Tumors of the skin and subcutaneous tissues. In: Vail, D.M., Thamm, D.H. & Liptak, J.M. Withrow & MacEwen’s Small Animal Clinical Oncology. 6^th^ Edition, Elsevier, St. Louis, Missouri, 2020, p. 357-358.

20. Melzer, K., Guscetti, F., Bley, C.R., Sumova, A., Roos, M. & Kaser-Hotz. Ki67 reactivity in nasal and periocular squamous cell carcinomas in cats treated with electron beam radiation therapy. *J. Vet. Intern. Med*. 20, 676–681 (2006).

21. Hunt, G.B. Use of the lip-to-lid flap for replacement of the lower eyelid in five cats. *Vet. Surg*. 35, 284–286 (2006).

22. Lana, S.E. *et al*. Feline cutaneous squamous cell carcinoma of the nasal planum and the pinnae: 61 cases. *J. Am. Anim. Hosp. Assoc*. 33, 329-332 (1997).

23. Pinard, C.L., Mutsaers, A.J., Mayer, M.N. & Woods, J.P. Prospective study and review of ocular radiation side effects following external-beam Cobalt-60 radiation therapy in 37 dogs and 12 cats. *Can. Vet. J.* 53, 1301–1307 (2012).

24. Cunha, S. C. S. *et al.* Radiation therapy for feline cutaneous squamous cell carcinoma using a hypofractionated protocol*. J. Fel. Med. Surg.* 12, 306-313 (2010).

25. Bexfield, N.H., Stell, A.J., Gear, R.N. & Dobson, J.M. Photodynamic therapy of superficial nasal planum squamous cell carcinomas in cats: 55 cases. *J. Vet. Intern. Med*. 22, 1385–1389 (2008).

26. Spugnini, E.P. et al. Intraoperative versus postoperative electrochemotherapy in high grade soft tissue sarcomas: a preliminary study in a spontaneous feline model. *Cancer Chemother. Pharmacol*. 59, 375-381 (2007).

27. Spugnini, E.P. et al. Adjuvant Electrochemotherapy for the Treatment of Incompletely Excised Spontaneous Canine Sarcomas. *In Vivo*. 21, 819-822 (2007).

28. Torrigiani, F., Pierini, A., Lowe, R., Simcic, P. & Lubas, G. Soft Tissue Sarcoma in Dogs: A Treatment Review and a Novel Approach Using Electrochemotherapy in a Case Series. Vet. Comp. Oncol. 17, 234-241 (2019).

29. Spugnini, E.P., Vincenzi, B., Amadio, B. & Baldi, A. Adjuvant electrochemotherapy with bleomycin and cisplatin combination for canine soft tissue sarcomas: A study of 30 cases. *Open Vet. J*. 9, 88-93 (2019).

30. Spugnini, E.P., Vincenzi, B., Carocci, F., Bonichi, C., Menicagli, F. & Baldi, A. Combination of bleomycin and cisplatin as adjuvant electrochemotherapy protocol for the treatment of incompletely excised feline injection-site sarcomas: A retrospective study. *Open Vet. J*. 10, 267-271 (2020).

31. Mali, B. *et al*. Tumor size and effectiveness of electrochemotherapy. Radiol. Oncol. 47, 32-41 (2013).

32.Clover, A.J.P. *et al*. Electrochemotherapy in the treatment of cutaneous malignancy: Outcomes and subgroup analysis from the cumulative results from the pan-European International Network for Sharing Practice in Electrochemotherapy database for 2482 lesions in 987 patients (2008–2019). *European J Cancer.* 138, 30-40 (2020).

33. Tozon, N., Kodre, V., Sersa, G. & Cemazar, M. Effective Treatment of Perianal Tumorsin Dogs with Electrochemotherapy. *Anticancer Res.* 25, 839-846 (2005).

34. Meads, M.B., Hazlehurst, L.A. & Dalton, W.S. The Bone Marrow Microenvironment as a Tumor Sanctuary and Contributor to Drug Resistance. *Clin. Cancer Res.* 14, 2519-2526 (2008).

35. David, E. *et al.* The Bone Niche of Chondrosarcoma: A Sanctuary for Drug Resistance, Tumour Growth and also a Source of New Therapeutic Targets. *Sarcoma*. 2011, 1-8 (2011).

36. Dos Anjos, D. *et al.* Electrochemotherapy induces tumor regression and decreases the proliferative index in canine cutaneous squamous cell carcinoma. *Sci. Reports*. 9, 15819 (2019).

37. National Research Council. Guide for the Care and Use of Laboratory Animals. Washington, National Academies Press, 8th edition (2011).

38. Campana, L. G. *et al*. Recommendations for improving the quality of reporting clinical electrochemotherapy studies based on qualitative systematic review. *Radiol. Oncol*. 50, 1–13 (2016).

39. Cemazar, M., Sersa, G., Frey, W., Miklavic, D. & Teissié, J. Recommendations and requirements for reporting on applications of electric pulse delivery for electroporation of biological samples. *Bioelechem.* 122, 69-76 (2018).

40. Owen, L. N. TNM Classification of tumor in domestic animals, World Health Organization (1980).

41. Gehl, J. *et al.* Updated standard operating procedures for electrochemotherapy of cutaneous tumours and skin metastases. *Acta Oncol*. 57, 874-882 (2018).

42. Tozon, N., Tratar, U.L., Znidar, K., Sersa, G., Teissie, J. & Cemazar, M. Operating Procedures of the Electrochemotherapy for Treatment of Tumor in Dogs and Cats. *J Vis Exp*. 116, 54760 (2016).

43. Eisenhauer, E.A. *et al.* New response evaluation criteria in solid tumours: Revised RECIST guideline (version 1.1). *Eur. J. Cancer*. 45, 228-247 (2009).

44. Nguyen, S.M., Thamm, D.H., Vail, D.M. & London, C.A. Response evaluation criteria for solid tumours in dogs (v1.0): a Veterinary Cooperative Oncology Group (VCOG) consensus document. *Vet. Comp. Oncol*. 13, 176-83 (2015).

Author Contributions:

Conceptualization, D.S.A.; O.R.S.; E.P.S.; A.B.N.; and C.E. FA.; methodology, D.S.A.; O.R.S.; E.P.S.; A.B.N.; and C.E. FA.; formal analysis, D.S.A.; E.P.S.; A.B.N., and C.E. FA.; investigation, D.S.A.; A.B.N.; and C.E. FA.; writing—original draft preparation, D.S.A.; O.R.S.; E.P.S.; A.B.N.; and C.E. FA.; statistical analysis, C.E. FA; preparation of the figures, tables and supplementary data, D.S.A. All authors reviewed the manuscript.

Conflicts of interest:

The authors have no conflicts of interest to declare.
