# Supplementary material for: Publisher Correction: Comparison of two different doses of bleomycin in electrochemotherapy protocols for feline cutaneous squamous cell carcinoma nonsegregated from ultraviolet light exposure
Source: Sci Rep. 2021 May 3;11:9759. doi: 10.1038/s41598-021-88998-8 (PMC8093240; doi:10.1038/s41598-021-88998-8)
Supplement: Supplementary file 1 — Supplementary Information. [file 41598_2021_88998_MOESM1_ESM.pdf]

Comparison of two different doses of bleomycin in electrochemotherapy protocols for feline cutaneous squamous cell carcinoma nonsegregated from ultraviolet light exposure

Denner S. Dos Anjos[1*], Oscar R. Sierra[1], Enrico P. Spugnini[2], Andrigo B. De Nardi[†1], Carlos E. Fonseca-Alves[†*3,4]

1 Department of Veterinary Clinic and Surgery, São Paulo State University (UNESP), Jaboticabal, Brazil

2 Biopulse srl, Via Toledo 256, Naples 80132, Italy

3 Department of Veterinary Surgery and Animal Anesthesiology, São Paulo State University - UNESP, Botucatu-SP, Brazil.

4 Institute of Health Sciences, Paulista University-UNIP, Bauru-SP, Brazil.

*Correspondence: denner.anjosoncology@gmail.com; +556798144-8191 and carlos.e.alves@unesp.br; +551499195-3871 (CEFA)

†These authors contributed equally to this work.

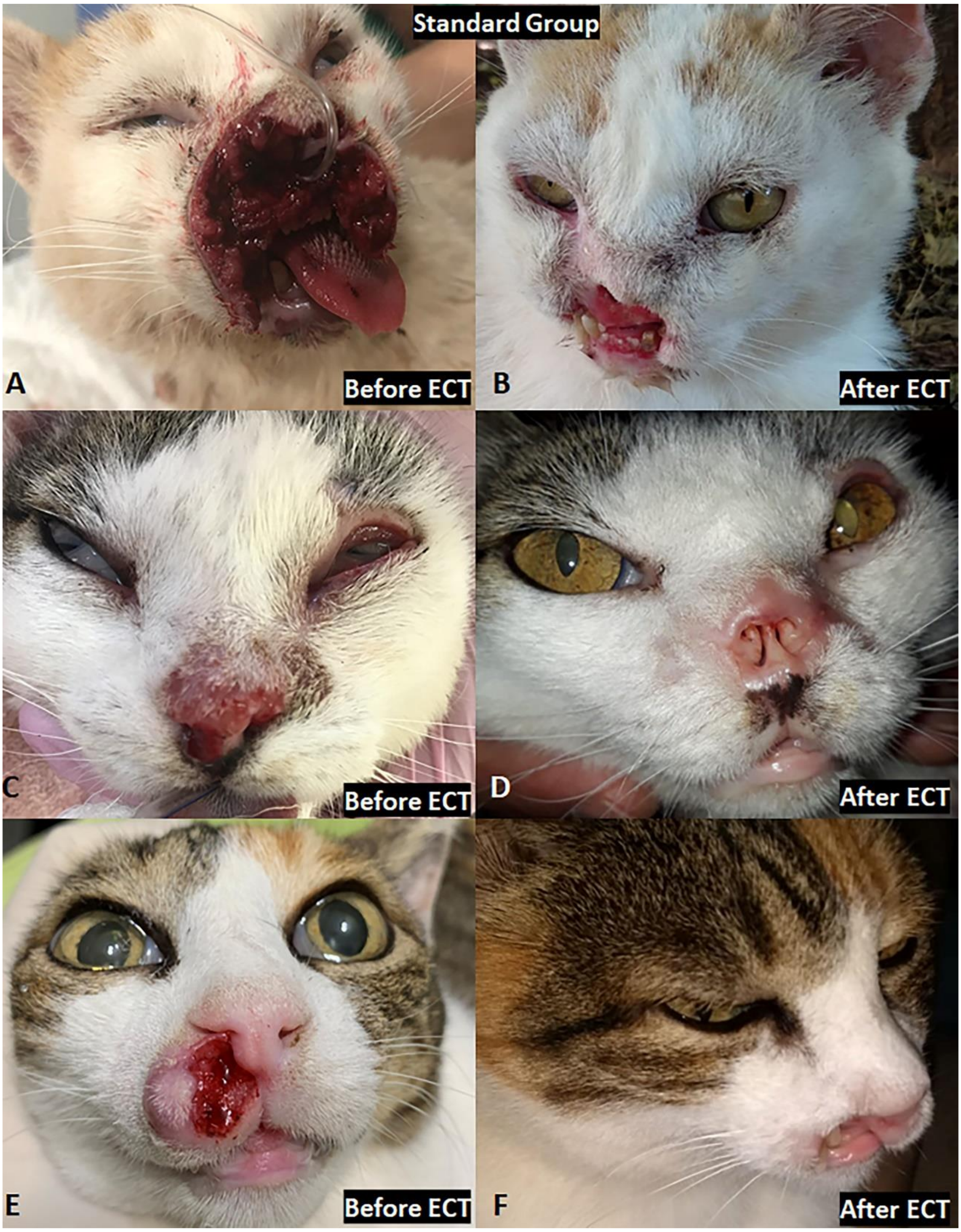

Supplementary figure 1. Cats diagnosed with cSCC subjected to ECT in standardized group (15,000 UI/m²). Patient 1 (A) with advanced carcinoma on the nose and upper lip prior ECT treatment and complete remission after two sessions (B). Patient 2 with cSCC on the nose, before ECT (C) and 30 days in complete remission after one session (D). Patient 3 with cSCC in upper right lip, before ECT (E) and 30 days in complete remission after one session (F).

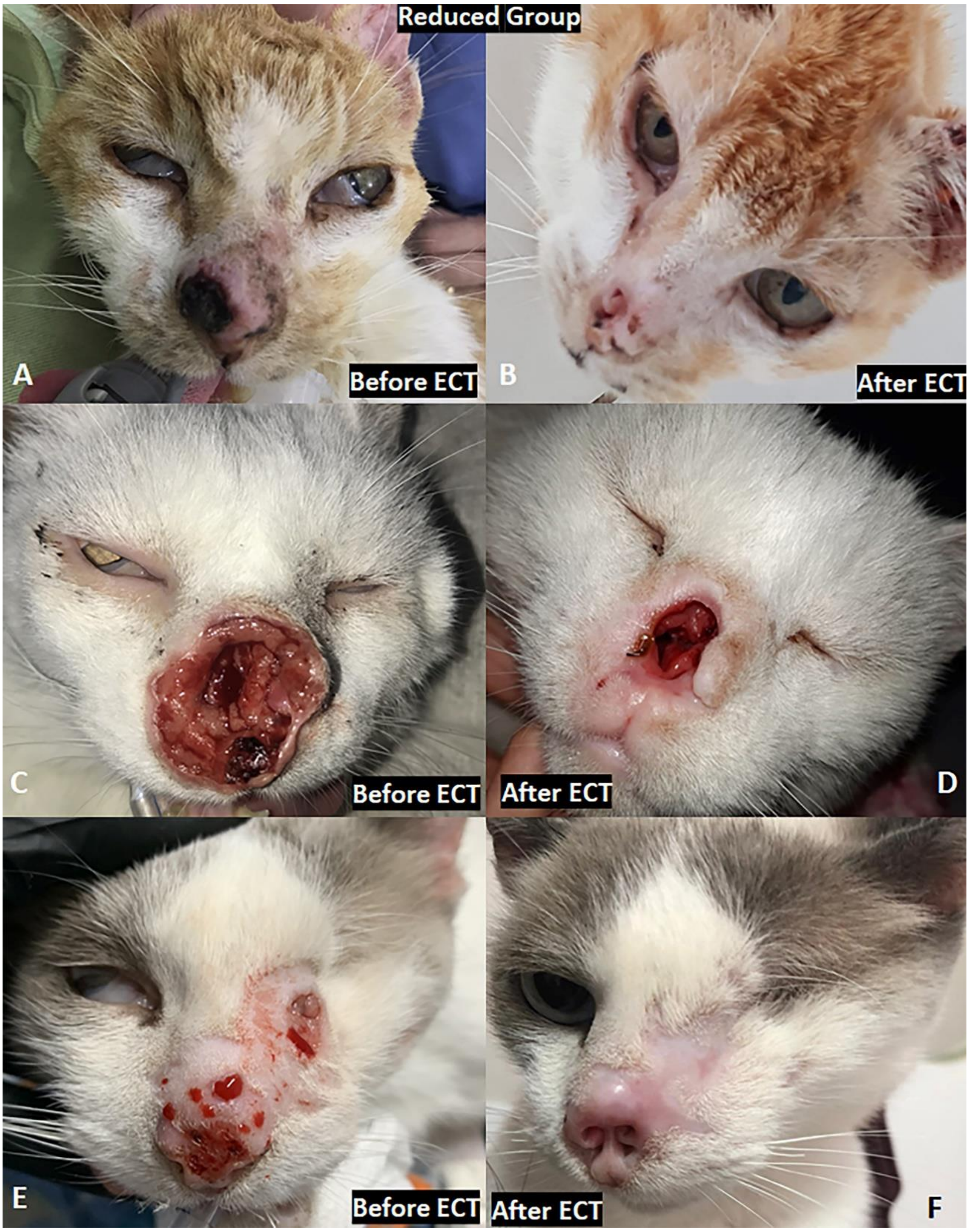

Supplementary figure 2. Cats diagnosed with cSCC subjected to ECT in reduced group (10,000 UI/m²). Patient 1 (A) with cSCC on the nose and prior ECT treatment and complete remission after one session (30 days) (B). Patient 2 with advanced carcinoma on the nose, before ECT (C) and 60 days in complete remission after one session (D). Patient 3 with cSCC on the nose and on left cutaneous side of the face, before ECT (E) and 30 days in complete remission after one session (F).

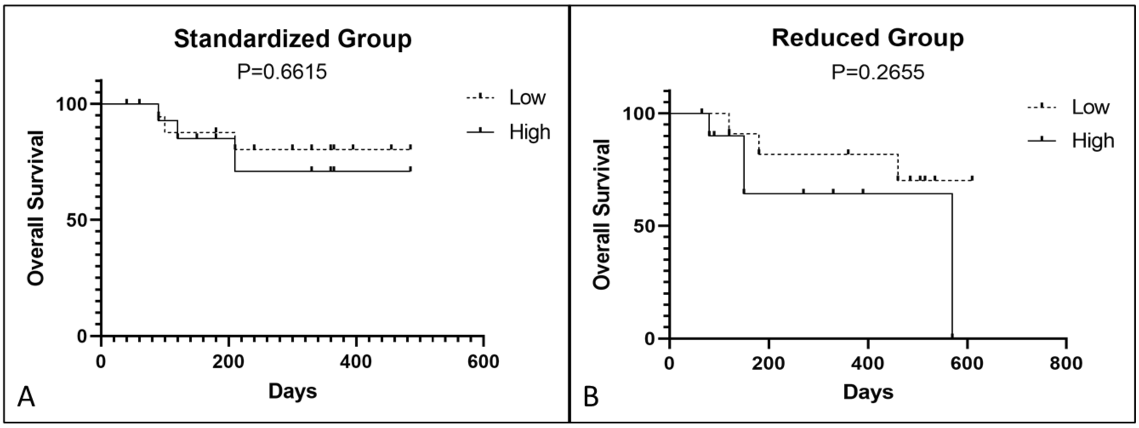

Supplementary figure 3. Kaplan-Meier curve for overall survival versus tumor size (low and high – cutoff 0.33cm³) between the 2 dose groups of cats with cSCC submitted to ECT. No statistically significant difference between the 2 doses groups was observed in the SG (P = 0.661) (A) nor RG (P = 0.265) (B).

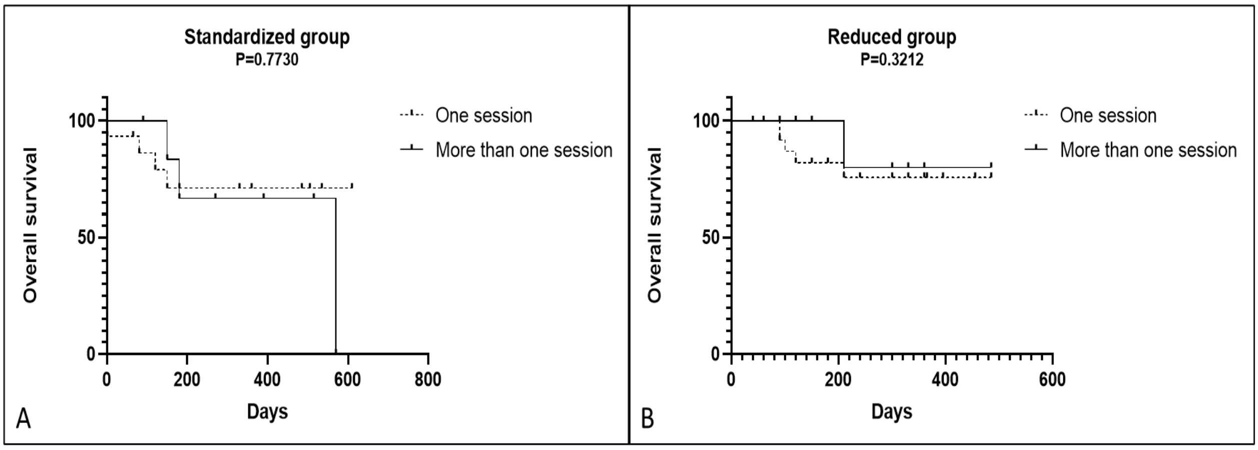

Supplementary figure 4. Kaplan-Meier curve for overall survival and number of ECT sessions between the 2 dose groups of cats with sCCC. No statistically significance difference was observed in survival among groups that received one or more than one session (P > 0.05).

Supplementary Table 1. Outcome of 66 lesions (56 cats) at 4 weeks post-ECT and at the end of observation period (45 lesions).

RECIST at 4 weeks	STANDARD DOSE				REDUCED DOSE			
**Variable**	**No(%)**	**CR (%)**	**PR (%)**	**SD (%)**	**No(%)**	**CR (%)**	**PR (%)**	**SD (%)**
**All lesions**	24	10	11	3	42	16	26	0
**Tumor size (cm)**								
**< 2**	16	9	5	2	30	13	17	0
**2-5 cm**	7	1	5	1	11	3	8	0
**> 5**	1	0	1	0	1	0	1	0
**End of observation period (1 - 20 months)**	**STANDARD DOSE**				**REDUCED DOSE**			
**Variable**	**No(%)**	**CR (%)**	**PD (%)**	**SD (%)**	**No(%)**	**CR (%)**	**PD (%)**	**SD (%)**
**All lesions**	14	10	3	1	29	21	5	3
**Tumor size (cm)**								
**< 2**	12	10	2	0	20	18	4	0
**2-5 cm**	2	0	1	1	8	5	0	3
**> 5**	0	0	0	0	1	0	1	0
